# PET/CT and Paraneoplastic Syndromes: A Comprehensive Review

**DOI:** 10.3390/cancers17162637

**Published:** 2025-08-13

**Authors:** Motaz Daraghma, Yashant Aswani, Sanchay Jain, Riccardo Laudicella, Ali Gholamrezanezhad, Yusuf Menda, Ahmad Shariftabrizi

**Affiliations:** 1 Saint Luke’s Hospital of Kansas City, University of Missouri-Kansas City, Kansas City, MO 64110, USA; motaz.daraghmeh@gmail.com; 2 Department of Radiology, University of Iowa Carver College of Medicine, Iowa City, IA 52242, USA; yashant-aswani@uiowa.edu (Y.A.); sanchay-jain@uiowa.edu (S.J.); yusuf-menda@uiowa.edu (Y.M.); 3 Nuclear Medicine Unit, Department of Biomedical, Dental Sciences and Morpho-Functional Imaging, Messina University, 98122 Messina, Italy; riccardo.laudicella@unime.it; 4 Department of Radiology, Keck School of Medicine, University of Southern California (USC), Los Angeles, CA 90089, USA; a.gholamrezanezhad@yahoo.com

**Keywords:** DOTATATE, FDG, metabolic imaging, occult malignancy detection, paraneoplastic symptoms, positron emission tomography

## Abstract

Paraneoplastic syndromes (PNSs) are pathologic conditions produced by neoplasms not attributable to tumor invasion or metastasis. The clinical manifestations of PNSs can precede the diagnosis; these symptoms may serve as early indicators of underlying malignancy. Standard imaging modalities, such as computed tomography (CT) and magnetic resonance imaging (MRI), have limited sensitivity in detecting small or early-stage PNS-associated tumors. FDG PET/CT identifies hypermetabolic lesions suggestive of malignancy and, therefore, facilitates early diagnosis, refined treatment planning, and potentially prolonged patient survival. This review evaluates the diagnostic accuracy, clinical utility, and emerging role of FDG PET/CT in detecting occult malignancies. Syndrome-targeted applications discussed include limbic encephalitis, cerebellar degeneration, Lambert-Eaton myasthenic syndrome, Cushing’s syndrome, hypercalcemia of malignancy, dermatomyositis, and tumor-induced osteomalacia. In addition, the limitations of FDG PET/CT, including false-positive or false-negative findings, are reviewed, while newer PET tracers, like ^68^Ga-DOTATATE, are also highlighted. Ultimately, FDG PET/CT has transformed clinical decision-making, enabling more timely interventions and improved patient management in the context of PNSs. Future directions in imaging, including PET/MRI and ongoing refinements in tracer design, promise to further enhance diagnostic precision, and therapeutic outcomes are also discussed.

## Simple Summary

Paraneoplastic syndromes are unusual medical conditions that occur as a result of cancer but are not caused by the direct spread of the tumor. These syndromes often affect the nervous system or other parts of the body and can appear before cancer is diagnosed. This review explores how a special imaging method called PET/CT can help detect hidden cancers linked to these syndromes. By identifying tumors early, doctors can start treatment sooner, which may improve patient outcomes. We discuss how PET/CT is used for different types of paraneoplastic syndromes and highlight its advantages and challenges. This summary is intended to help non-specialists understand the importance of PET/CT in uncovering cancers that might otherwise go unnoticed.

## 1. Introduction

Paraneoplastic syndromes (PNSs) consist of a heterogeneous spectrum of pathologic conditions occurring because of malignancy-related systemic effects but not resulting directly due to direct tumor invasion and/or metastatic spread. These syndromes develop as a result of immune responses against common tumor-neuronal antigens or abnormal release of mediators, such as hormones, cytokines, and peptides, which lead to multiple system malfunctions [1,2,3]. PNSs often appear before a malignancy diagnosis is confirmed, which makes them important potential indicators of occult malignancies [4,5].

Conventional imaging modalities, such as computed tomography (CT) and magnetic resonance imaging (MRI), have limited capabilities to identify early-stage or small tumors linked to PNSs. FDG PET/CT combines metabolic imaging with structural imaging to detect hypermetabolic lesions that show malignancy with higher diagnostic accuracy compared to other methods [6,7,8]. FDG PET/CT enables the early identification of occult malignancies, playing a crucial role in guiding diagnostic decisions and treatment planning [9,10].

While previous reviews have addressed the role of FDG PET/CT in PNSs, they have largely focused on its established oncologic applications and have given limited attention to recent advances. The present work expands upon earlier literature by incorporating developments in complementary tracers, such as [^68^Ga]Ga-DOTATATE and hybrid PET/MRI, which have not been comprehensively discussed in prior summaries.

The relevant literature was identified through a targeted search of PubMed, Scopus, and Web of Science for articles published up to March 2025. Search terms included combinations of “paraneoplastic syndromes,” “FDG PET/CT,” “positron emission tomography,” “DOTATATE,” “DOTANOC,” “somatostatin receptor imaging,” “PET/MRI,” and “PET tracers.” Reference lists of relevant articles were manually screened to identify additional studies. Priority was given to peer-reviewed publications in English that reported on the diagnostic role, clinical utility, or illustrative case examples of PET imaging in PNSs. Editorials, conference abstracts without full text, non-peer-reviewed material, and studies not specifically addressing PET imaging in the context of PNSs were excluded.

This review evaluates the diagnostic accuracy, clinical utility, and potential of FDG PET/CT in detecting occult malignancies associated with PNSs, with a focus on syndrome-specific applications and patient outcomes.

## 2. FDG PET/CT Basics

**Figure 1:**
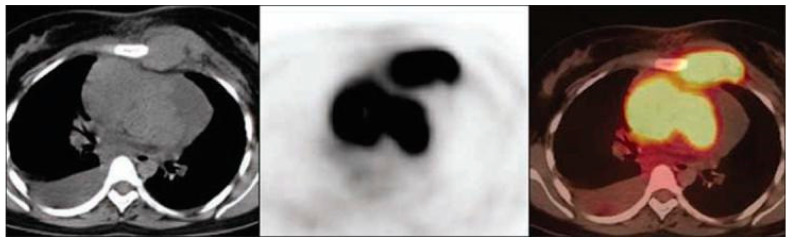
Axial images from non-contrast CT, PET, and PET-CT demonstrate elevated FDG uptake in the mediastinal and the left internal mammary lymph nodes in a patient diagnosed with lymphoma. Source: [17].

FDG PET/CT is important for molecular imaging modality in the management of various malignancies including for staging, restaging, treatment response evaluation, and detecting recurrence. It may also be useful for identifying the sites most amenable to biopsy. F-18 Fluorodeoxyglucose (FDG), is a radiolabeled glucose analog with Fluorine-18 that malignant cells absorb more than normal cells because these cancer cells consume glucose at higher rates [11,12]. Its uptake is mediated via glucose transporters (GLUTs), and after being internalized into the cells, FDG is phosphorylated but not metabolized, which leads to its intracellular accumulation linked to glycolytic activity levels [3,10,13]. This retention is visualized on a PET scan as a focal hypermetabolic region that matches neoplastic or inflammatory processes [12,14]. FDG PET/CT has the advantage of providing metabolic information in addition to the anatomical localization by the CT component over standalone imaging modalities (CT and MR imaging) [11,15,16]. As shown in Figure 1, FDG PET/CT effectively identifies hypermetabolic regions in mediastinal lymph nodes, demonstrating its utility in diagnosing malignancy-related systemic effects. The hybrid method increases diagnostic sensitivity for small and metabolically active tumors, for which standalone CT or MRI may have limited sensitivity [11,15,16].

The ability of FDG PET/CT to identify hypermetabolic lesions leads to incidental detection of PNSs during imaging intended for either detecting occult malignancy or even unrelated indications. Younes-Mhenni et al. published a study in which the FDG PET/CT identified sites of abnormal uptake in 18 out of 20 patients with suspected PNSs in whom the anatomic imaging was negative, and found cancer diagnoses in 14 of these patients [8]. Similarly, in the study published by Opalińska et al. [7], FDG PET/CT scans detected malignancies in 53% of patient evaluated for PNSs who had normal anatomic imaging, enabling earlier diagnosis. The metabolic ‘signature’ of FDG allows PET/CT to overcome diagnostic challenges in PNSs since immune mechanisms and hormone production precede physical tumor changes [7,8].

## 3. Classification of Paraneoplastic Syndromes

Paraneoplastic syndromes (PNSs) are classified as neurologic and non-neurologic. Different mechanisms involving the immune response(s) against tumor-neuronal antigens and release of tumor-produced substances such as hormones and cytokines, lead to these syndromes. FDG PET/CT serves as an essential diagnostic tool for PNSs because it detects occult tumors and evaluates metabolic changes linked to these syndromes [18,19,20].

### 3.1. Neurologic Paraneoplastic Syndromes

Neurologic PNSs are predominantly caused by immune-mediated mechanisms directed towards neuronal tissues due to antigenic similarity between tumor cells and neural structures. These shared antigens, termed onconeural antigens, trigger the production of autoantibodies, such as anti-Hu, anti-Yo, anti-Ri, and anti-Ma2, which can be detected in serum or cerebrospinal fluid (CSF) [2,3,9]. Neurologic manifestations are categorized into syndromes affecting the central nervous system (CNS) and peripheral nervous system (PNS). FDG PET/CT contributes critically to the identification of hypermetabolic malignancies, such as small-cell lung cancer [SCLC], breast cancer, and ovarian cancer [10,21,22].

Table 1 provides a summary of key neurologic paraneoplastic syndromes, including their associated antibodies, malignancies, and characteristic FDG PET/CT findings.

#### 3.1.1. Central Nervous System Syndromes

Paraneoplastic Limbic Encephalitis (PLE) causes inflammation in the limbic system, which controls memory functions alongside emotional and behavioral regulation. The pathophysiology involves autoantibodies (anti-Hu, anti-Ma2) targeting neuronal surface or intracellular antigens, leading to neuronal dysfunction and apoptosis [23,24,25]. Clinically, the patients may present with memory impairment, confusion, seizures, or behavioral disturbances [23,26]. SCLC is the most frequently associated malignancy, particularly in cases with anti-Hu antibodies, though thymoma and breast cancer have also been reported as the causes of PLE [23,27,28].

Paraneoplastic Cerebellar Degeneration (PCD), also known as Paraneoplastic Cerebellar Ataxia (PCA), manifests as a rapidly progressive cerebellar syndrome marked by ataxia, dysarthria, and nystagmus [25,29]. The pathophysiology of PCD involves antibody-mediated damage to Purkinje cells, and anti-Yo, anti-Tr, and anti-CV2 antibodies are strongly implicated [25,29,30]. Anti-Yo antibodies are specifically associated with ovarian and breast cancers.

Opsoclonus-Myoclonus Syndrome (OMS) is characterized by involuntary, multidirectional eye movements (opsoclonus) and jerking limb movements (myoclonus) [31]. The pathophysiology involves both humoral and cell-mediated immune responses against neuronal antigens, and are associated with anti-Hu, anti-Ri, and anti-SOX1 antibodies [32,33,34]. In pediatric populations, OMS is strongly associated with neuroblastomas, whereas adults commonly present with underlying SCLC or breast cancer [33,35,36,37,38,39].

#### 3.1.2. Peripheral Nervous System (PNS) Syndromes

Lambert-Eaton Myasthenic Syndrome (LEMS) is a neuromuscular junction (NMJ) disorder characterized by proximal muscle weakness and autonomic dysfunction [40,41]. The pathophysiology involves autoantibodies targeting presynaptic voltage-gated calcium channels (VGCCs), impairing acetylcholine release at the NMJ [40,41]. LEMS is strongly associated with SCLC, most mediated by anti-VGCC antibodies [40,41].

Subacute Sensory Neuronopathy (SSN) presents with progressive sensory loss, often beginning in the lower extremities [42,43,44]. The pathophysiology involves an autoimmune attack on dorsal root ganglia neurons, mediated predominantly by anti-Hu antibodies [42,45,46]. SCLC is the most common underlying malignancy [47,48].

### 3.2. Non-Neurologic Paraneoplastic Syndromes

Non-neurologic PNS arise primarily from the ectopic secretion of tumor-derived bioactive substances, including hormones, cytokines and peptides [2]. These syndromes affect primarily endocrine, dermatologic, musculoskeletal, hematologic, and gastrointestinal systems [2]. A summary of key non-neurologic paraneoplastic syndromes, including their underlying mechanisms, associated malignancies, and the role of FDG PET/CT, is provided in Table 2.

#### 3.2.1. Endocrinologic Syndromes

Paraneoplastic Cushing’s syndrome occurs as a result of excessive cortisol production due to ectopic Adrenocorticotropic Hormone (ACTH) release from non-pituitary tumors. Malignancies, including small-cell lung cancer (SCLC), thymic carcinomas, and pancreatic neuroendocrine tumors, release ACTH, which causes adrenal hyperplasia resulting in excessive cortisol production [49,50,51,52], which most commonly leads to weight gain, moon facies, truncal obesity, hypertension, and hyperglycemia [53,54].

Hypercalcemia of malignancy results from tumor-produced parathyroid hormone-related protein (PTHrP) which simulates the actions of parathyroid hormone (PTH) and leads to bone loss and kidney calcium retention [55,56,57,58]. Commonly associated malignancies include breast cancer, lung cancer, renal cell carcinoma, and multiple myeloma [55,59,60,61]. Fatigue, anorexia, nausea, and polyuria make it challenging to distinguish primary hyperparathyroidism from other conditions [59,60,61].

#### 3.2.2. Dermatological Syndromes

Dermatomyositis is an autoimmune inflammatory myopathy characterized by proximal muscle weakness, heliotrope rash (a violet or dusky discoloration of the eyelids), and Gottron’s papules (raised, scaly, violaceous eruptions typically found over knuckles and joints). The pathophysiology involves tumor-associated antigens triggering antibodies such as anti-TIF1-γ and anti-NXP2, with strong association with lung, ovarian, and gastrointestinal malignancies [62,63,64,65].

Acanthosis nigricans presents as hyperpigmented, velvety plaques in intertriginous areas, which is driven by tumor overproduction of transforming growth factor-alpha (TGF-α) and insulin-like growth factors (IGFs) stimulating epidermal proliferation [66,67,68,69]. This syndrome is strongly linked to gastrointestinal malignancies, particularly gastric adenocarcinoma [70,71,72].

Paraneoplastic pemphigus, a severe autoimmune blistering disorder, manifests with mucocutaneous erosion, which is associated with lymphoproliferative malignancies, such as non-Hodgkin’s lymphoma and Castleman disease [73,74]. Autoantibodies targeting desmosomal proteins, such as anti-envoplakin and anti-desmoplakin, induce acantholysis and epithelial detachment [75,76,77].

#### 3.2.3. Musculoskeletal Syndromes

Tumor-induced osteomalacia (TIO), or oncogenic osteomalacia, results from excessive fibroblast growth factor 23 (FGF23) secretion by phosphaturic mesenchymal tumors (PMTs), leading to renal phosphate wasting and hypovitaminosis D [78,79,80]. Patients experience chronic bone pain, muscle weakness, and pathologic fractures [79,81,82].

Hypertrophic osteoarthropathy (HOA) is characterized by digital clubbing, periosteal new bone formation, and arthralgia, commonly associated with lung and gastrointestinal malignancies [83,84,85,86,87]. Vascular endothelial growth factor (VEGF) and other tumor-derived factors are implicated in periosteal proliferation and vascular remodeling [88,89].

#### 3.2.4. Rheumatological Syndromes

Paraneoplastic vasculitis is an immune-mediated inflammation of blood vessels that arises secondary to an underlying malignancy triggered by tumor antigens, immune complex deposition, or cytokine dysregulation [90,91,92,93]. It often presents with cutaneous signs, such as palpable purpura or petechiae, but may also lead to neuropathies or organ ischemia, especially in systemic forms [90,91,92,93]. This syndrome has been observed in both solid tumors, such as gastric adenocarcinoma, renal cell carcinoma, and non-small cell lung carcinoma, and hematologic malignancies, including lymphoma, acute lymphoblastic leukemia (ALL), and chronic lymphocytic leukemia (CLL) [90,91,92,93]. In many cases, vasculitis resolves with successful cancer treatment and flares in parallel with tumor progression, strongly supporting its classification as a true paraneoplastic syndrome [90,91,92,93].

#### 3.2.5. Hematologic Syndromes

Paraneoplastic polycythemia arises from ectopic erythropoietin (EPO) production, most commonly by renal cell carcinoma or cerebellar hemangioblastomas, leading to elevated red blood cell mass and hyperviscosity [94].

Paraneoplastic thrombocytosis results from interleukin-6 (IL-6)-mediated thrombopoiesis observed in ovarian, lung, and gastrointestinal cancers, which increases the thromboembolic risk [1,95].

Paraneoplastic leukemoid reaction (PLR) is marked by extreme leukocytosis (>50,000/µL) due to tumor secretion of granulocyte colony-stimulating factor (G-CSF) or granulocyte-macrophage colony-stimulating factor (GM-CSF). It is associated with non-small cell lung cancer (NSCLC) and inflammatory liposarcoma, often indicating aggressive tumor behavior [96,97,98,99,100,101].

#### 3.2.6. Gastrointestinal Syndromes

Paraneoplastic diarrhea (VIPoma syndrome) is caused by vasoactive intestinal peptide (VIP) secretion from pancreatic neuroendocrine tumors (VIPomas) or medullary thyroid carcinoma, resulting in secretory diarrhea, hypokalemia, and dehydration [102,103].

## 4. Role of PET/CT in Paraneoplastic Syndromes

The diagnosis of paraneoplastic syndromes (PNSs) can be challenging because of their varied clinical presentations and the occult nature of underlying malignancies. Treatment strategy and improved patient outcomes depend on the identification of the associated malignancy. FDG PET/CT has emerged as a valuable diagnostic tool for identifying or excluding occult malignancies in patients with suspected paraneoplastic syndromes [104].

### 4.1. Paraneoplastic Limbic Encephalitis (PLE)

**Figure 2:**
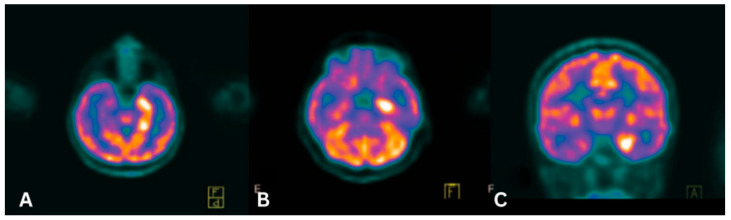
(**A**,**B**) Axial and (**C**) coronal: FDG PET/CT image shows significant asymmetric hypermetabolism of the bilateral mesial temporal lobes, consistent with limbic encephalitis. Clinical takeaway: PET/CT can reveal metabolic changes supporting PLE even when anatomic imaging is equivocal. Source [19].

In PLE, FDG PET/CT demonstrates increased metabolic activity in the medial temporal lobes of PLE patients, particularly in the hippocampus and amygdala, (Figure 2A–C) [23,27,105]. A systematic review of 176 studies involving 720 patients found an FDG PET sensitivity of 90%, whereas another meta-analysis covering 21 studies with 444 patients showed a sensitivity of 80% [106]. Although PET/CT demonstrates significant diagnostic capabilities, it has notable limitations that should be considered.

The primary diagnostic challenge for PLE stems from its subtle or variable metabolic presentation on PET/CT, which may manifest as mild, localized, or asymmetric hypermetabolism in the mesial temporal lobes that can be overlooked or misinterpreted, especially in early disease stages or antibody-negative cases, making diagnosis with only standard imaging more challenging [107,108]. In such cases, the integration of PET/MRI improves diagnostic precision by combining metabolic data with information regarding structural changes more apparent on MR sequences, which enhances localization accuracy and minimizes diagnostic errors [107]. Moreover, the image interpretation faces challenges from either false positive findings, such as inflammatory lesions or benign hypermetabolic foci that may mimic malignancies, or false negatives, including tumors that do not demonstrate marked hypermetabolism or are below the resolution threshold of PET/CT [107,108,109]. To address these challenges, it is imperative to use serial PET/CT scans along with onconeural antibody tests and multimodal imaging strategies to enhance diagnostic accuracy and early clinical interventions [108].

### 4.2. Paraneoplastic Cerebellar Degeneration (PCD)

In PCD, anatomic neuroimaging is usually unremarkable in the early stages, making metabolic imaging particularly valuable [110]. FDG PET-CT findings in PCD may show cerebellar hypermetabolism in acute stages, potentially evolving into hypometabolism in chronic phases, [111]. A recent case report documented cerebellar hypermetabolism on FDG PET/CT in a patient with anti-Yo antibody-positive PCD despite unremarkable MRI findings, highlighting the added diagnostic value of metabolic imaging [111].

Clinical integration of PET/CT with antibody testing and MRI significantly increases diagnostic yield [111]. Even in seronegative cases, brain FDG PET/CT can accurately disclose the longitudinal pathologic changes of brain metabolism occurring in acute and post-treatment remission stages, paralleling clinical impairment and response to treatment [112]. MRI may eventually show cerebellar atrophy, but PET can identify metabolic abnormalities even before the structural changes become evident [113]. Only one of four patients with PCD in one study had reduced cerebellar uptake on FDG-PET, which was associated with cerebellar vermis atrophy on MRI, indicating heterogeneous metabolic phenotypes [113]; therefore, caution should be used when interpreting findings on PET. Furthermore, infections or other inflammatory processes affecting cerebellar metabolism may yield false positives [114]. Despite these limitations, FDG PET/CT remains a valuable tool in guiding the diagnosis of PCD, especially when conventional imaging fails to detect the underlying malignancy [111,112].

### 4.3. Opsoclonus-Myoclonus Syndrome (OMS)

**Figure 3:**
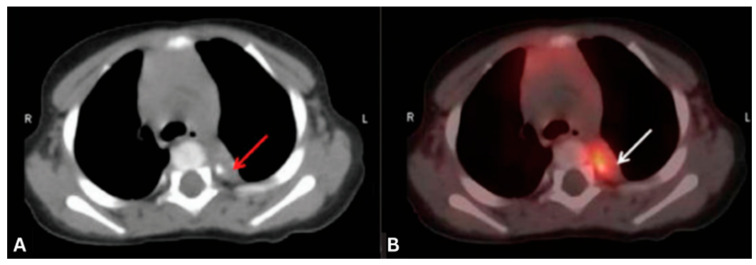
(**A**) Axial non-contrast CT image and (**B**) PET/CT images show hypermetabolic left paravertebral soft tissue with calcification (arrow) in pediatric OMS proven to be neuroblastoma on histopathology. Clinical takeaway: Demonstrates PET/CT’s ability to localize metabolically active neuroblastic tumors in OMS, enabling definitive diagnosis and guiding timely treatment. Source [116].

In OMS, FDG PET/CT reveals thoracic or breast malignancies. PET/CT also identifies cerebellar or brainstem metabolic changes indicative of neuroinflammation (Figure 3) [36,115,116]. ^68^Ga-DOTATATE PET/CT serves as an important complementary tool for imaging neuroendocrine tumors like neuroblastomas in children, which exhibit high somatostatin receptor expression, enhancing detection sensitivity [115,117]. [115]. This result came from a pediatric OMS cohort (n = 38) with a high neuroblastic tumor prevalence (~45%), which should be considered when interpreting the 100% NPV [115]. These findings demonstrate PET/CT’s diagnostic accuracy, particularly when MRI and MIBG scintigraphy yield inconclusive results.

Despite its utility, somatostatin receptor (SSR) PET/CT with ^68^Ga-DOTATATE has some limitations in OMS detection. False positives and false negatives have been reported, as demonstrated by cases where reactive hyperplasia of the adrenal gland was misinterpreted as a tumor, which can lead to diagnostic errors [21,115]. Another limitation is the relative lack of large-scale validation studies available for [115,116]. Additionally, PET/CT accuracy varies based on disease stage and tumor characteristics [118,119]. Furthermore, PET/CT has reduced diagnostic performance in detecting non-neuroblastic tumors associated with OMS, such as mature cystic teratomas and small-cell lung cancer, which can result in missed diagnoses [120].

### 4.4. Lambert-Eaton Myasthenic Syndrome (LEMS)

**Figure 4:**
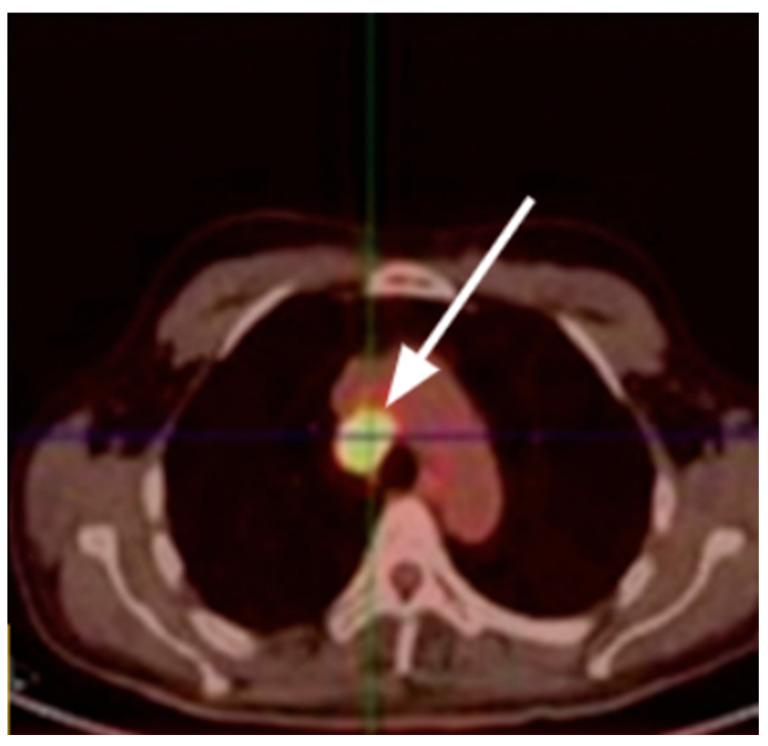
Axial PET/CT demonstrates hypermetabolic mediastinal lymphadenopathy (arrow) in LEMS. Clinical takeaway: Highlights PET/CT’s role in detecting thoracic malignancy—often small-cell lung cancer—as the underlying cause of LEMS, enabling early diagnosis and targeted management. Source [127].

FDG PET/CT detects thoracic malignant tumors that typically demonstrate high FDG uptake, specifically SCLC, which remains the most frequent tumor type associated with LEMS (Figure 4) [121,122,123,124]. LEMS patients may need long-term monitoring and repeated imaging to identify the presence of the malignancy as very early stage may still be undetectable by PET/CT [125,126]. In some cases, PET/CT identifies extrapulmonary tumors, such as testicular or cerebral metastases, which may initially mislead clinicians or obscure the detection of the underlying primary malignancy, as seen in small-cell lung cancer or prostate cancer recurrences. This can complicate diagnosis and delay appropriate treatment [123,124].

Given these challenges, PET/CT is used in conjunction with other imaging techniques, such as CT-thorax, which is frequently utilized to enhance detection rates [123]. For instance, in the study by Titulaer et al., CT-thorax detected SCLC in 83% of cases [123]. However, FDG PET/CT offers complementary value by identifying additional malignancies not visualized on CT scans and by providing superior whole-body staging, particularly for metastatic disease. In the same cohort, PET/CT detected occult lesions and clarified equivocal CT findings, supporting its use as an adjunct rather than a replacement for CT in the diagnostic workup and staging of suspected paraneoplastic SCLC. Additionally, delayed tumor detection remains a challenge because some SCLC cases are not identifiable on PET/CT imaging for even months following the initial LEMS diagnosis. Continuous imaging follow-ups are essential for patients with SOX-1 antibodies, to identify occult SCLC [125,126]. While PET/CT improves the detection of malignancies associated with LEMS, clinicians should understand its early-stage detection limits and false-positive potential, such as inflammatory lesions, benign nodules, or reactive lymph nodes that can mimic malignancy, and combine FDG PET/CT with other imaging methods for better diagnostic accuracy.

### 4.5. Paraneoplastic Cushing’s Syndrome (PCS)

**Figure 5:**
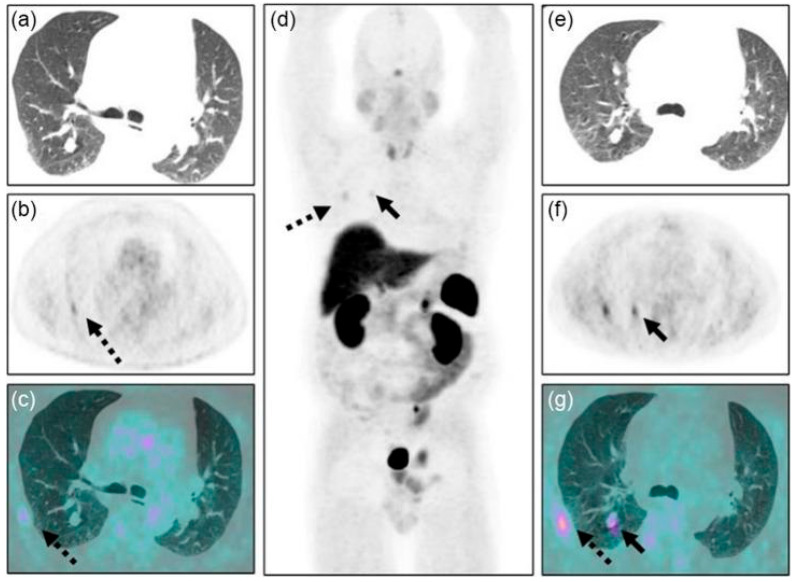
A 58-year-old man with ectopic ACTH-secreting Cushing’s syndrome. FDG PET/CT images (**a**–**c**) show a non-avid lung nodule. MIP, trans-axial CT, PET, and fused PET/CT images (**d**–**g**) show mild focal [[^68^Ga68Ga]Ga-DOTA-TATE uptake (SUVmax 1.9) in a right lower lobe nodule (solid black arrows). Post-surgery histological diagnosis confirmed a typical bronchial carcinoid secreting ACTH. Uptake is also seen with FDG (**b**,**c**) and [[^68^Ga68Ga]Ga-DOTA-TATE (**d**,**f**,**g**) PET/CT due to a rib fracture (black dashed arrows). Clinical takeaway: Demonstrates the complementary role of targeted tracers like [^68^Ga]Ga-DOTA-TATE in localizing low-FDG-avid neuroendocrine tumors in PCS and highlights the importance of recognizing benign causes of tracer uptake such as fractures. Source: [135].

FDG PET/CT imaging is vital for finding ectopic ACTH-producing tumors in paraneoplastic Cushing’s syndrome (PCS) patients, especially when these tumors are in the pancreas, thymus, or lungs, as illustrated in Figure 5. The accurate localization of these tumors remains essential for guiding subsequent medical or surgical interventions [128]. In tumors that inherently do not demonstrate significant FDG uptake, receptor-specific PET tracers may be utilized, such as ^68^Ga-DOTATATE/-DOTATOC for well-differentiated neuroendocrine tumors linked to PCS. Compared to traditional 111In-octreotide, these newer [129,130]. A systematic review of 33 studies assessing [131]. This limitation is clinically important, as physiologic or hyperplastic adrenal uptake can mimic metastatic or primary adrenal tumors in PCS. For instance, bilateral adrenal hyperplasia in ectopic ACTH syndrome may present with intense uptake, potentially obscuring small metastatic foci. Differentiation can be improved by adrenal CT washout analysis (absolute washout ≥ 60% or relative ≥ 40%), MRI chemical shift imaging, and biochemical correlation (e.g., cortisol, ACTH). In ambiguous cases, follow-up imaging or biopsy may be warranted before intervention [132,133,134].

The diagnostic accuracy of PET/CT in PCS remains variable, with inconsistent sensitivity and specificity observed in different studies. ^68^Ga-SSTR PET/CT shows high sensitivity in certain cases but achieves only 64% sensitivity on average, which presents a risk of false negatives [131,136]. Additionally, false positive and false negative results continue to be problematic when benign adrenal uptake leads to tumor misdiagnosis or when actual tumors are not detected because of low metabolic activity [136]. The selection of PET tracers represents a critical challenge that influences the accuracy of diagnostic results. Studies indicate that [^18^F]FDOPA and [^68^Ga]Ga-DOTANOC have superior detection capabilities compared to [^18^F]FDG for ACTH-secreting tumors, but the best tracer selection for PCS diagnosis continues to be unclear, which may result in diagnostic dilemma [137]. Moreover, the current evidence for [131].

### 4.6. Hypercalcemia of Malignancy

**Figure 6:**
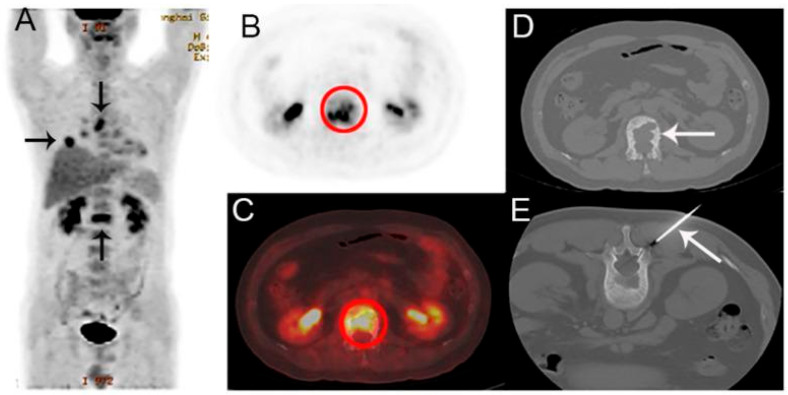
A 44-year-old man presented with back pain, suspected of having lung cancer with bone metastasis based on PET/CT. (**A**) Coronal maximum intensity projection (MIP) FDG PET image shows multiple FDG-avid lesions in the lung, a mediastinal lymph node, and L1 (arrows). (**B**,**C**) Axial FDG PET image (**B**) and fusion images (**C**) demonstrate an FDG-avid lesion (SUVmax 6.7) in L1 (circle) (**D**,**E**) with corresponding axial CT (**D**) and CT-guided biopsy images (**E**). Histological examination confirmed that the bone lesion was metastatic lung adenocarcinoma. EGFR and ALK were detected as wild type and negative, respectively. Clinical takeaway: Highlights PET/CT’s role in detecting and staging metastatic lung cancer, directing biopsy to metabolically active lesions for definitive diagnosis and guiding targeted molecular testing. Source: [139].

FDG PET/CT is capable of detecting both hypermetabolic tumors that secrete PTHrP and lytic bone metastases (Figure 6), FDG PET/CT imaging shows high sensitivity and specificity when detecting cancers that result in hypercalcemia. It achieves a 99% sensitivity rate in detecting bone metastases, which surpasses the 87% sensitivity of standalone CT and proves to be an accurate and reliable diagnostic tool [138]. Additionally, PET/CT shows a specificity of 95%, compared to 93% for standalone CT [138].

### 4.7. Dermatomyositis (DM)

FDG PET/CT detects occult malignancies and differentiates paraneoplastic dermatomyositis from idiopathic forms by identifying hypermetabolic tumors and inflammatory muscle involvement [140,141,142,143]. The use of FDG PET/CT for tumor detection shows a positive predictive value (PPV) of 85.7%, a negative predictive value (NPV) of 93.8%, a sensitivity of 66.7%, a specificity of 97.8%, and an overall predictive value of 92.7% [143]. Additionally, PET/CT imaging is effective in monitoring disease progression in DM-associated interstitial lung disease, with sensitivity and specificity levels of 77.8% and 72.8%, respectively [144]. These results indicate that PET/CT serves as a valuable imaging tool for both tumor detection and assessment of inflammatory disease activity in DM cases.

PET/CT in dermatomyositis cases presents several challenges. False positives due to inflammatory conditions, such as ulcerative colitis or sacroiliitis, may lead to diagnostic errors that result in unnecessary additional procedures [145]. The lack of standardized interpretation criteria for PET/CT in dermatomyositis contributes to inconsistent diagnosis and evaluation of cancer presence and disease activity [146]. Additionally, cost-effectiveness and radiation exposure continue to be major factors in patient evaluation. According to Kundrick et al., PET/CT is more economical compared to conventional cancer screening methods but costs healthcare systems and insurers more [147]. However, cost remains a practical barrier in some healthcare systems, and global accessibility is uneven. While FDG PET/CT is widely available, access to specialized tracers such as ^68^Ga-DOTATATE may be limited in low-resource settings due to production and infrastructure constraints [143,147]. In such contexts, a pragmatic strategy may involve FDG PET/CT combined with targeted anatomic imaging, reserving SSTR-targeted PET for cases with high suspicion where referral to specialized facilities is possible. Its reduced sensitivity for detecting certain cancers linked to DM highlights the need for complementary diagnostic approaches [140]. PET/CT findings have shown significant correlations between FDG uptake (SUVmax) and disease activity markers, such as serum KL-6 levels in the lungs and serum creatine kinase and aldolase levels in muscle tissue, suggesting the potential role of PET/CT scans in assessing inflammatory burden in DM patients, although its capability to completely represent disease spread needs further research [142,148].

### 4.8. Tumor-Induced Osteomalacia (TIO)

FDG PET/CT and somatostatin receptor-based PET tracers serve as valuable tools for identifying phosphaturic mesenchymal tumors (PMTs) that cause tumor-induced osteomalacia (TIO) [149,150,151,152,153]. Similarly, ^18^F]-AlF-NOTA-octreotide PET/CT has shown an 87.5% sensitivity and a 100% specificity rate that reinforces its application in tumor localization and patient management [154,155]. Among available tracers, ^68^Ga-DOTATATE PET/CT stands out among multiple tracers because of its 95.13% sensitivity and 60.00% specificity values, which make it the top choice for TIO imaging when standard methods prove ineffective [151,152]. Additionally, [156,157]. A comparative study showed that [158]. Additionally, ^68^Ga-DOTATATE PET/CT shows higher sensitivity and specificity compared to Octreoscan-SPECT/CT and ^18^F]-FDG PET, which makes it the preferred choice for locating PMTs in TIO [159]. PET/CT sometimes produces false positives and negatives, which result in misinterpretations of non-tumor tissue areas in TIO. For instance, in a 45-year-old woman with suspected TIO, [^68^Ga]Ga-DOTATATE PET/CT revealed focal uptake in the second right rib; however, biopsy results did not confirm malignancy, highlighting the risk of false positives due to uptake in healing fractures or benign lesions [152]. Conversely, PET/CT scans may fail to identify certain tumors owing to their low metabolic activity and result in false negatives [152]. Another major challenge is that advanced PET/CT imaging techniques face major detection issues when identifying small tumors or those with low metabolic activity [153,160].

### 4.9. Other Paraneoplastic Syndromes with Limited PET/CT Data

FDG PET/CT and receptor-specific PET tracers have demonstrated utility in evaluating various paraneoplastic syndromes, although available data on sensitivity, specificity, and limitations remain sparse.

In hypertrophic osteoarthropathy (HOA), FDG PET/CT imaging plays a role in detecting hypermetabolic primary tumors, particularly lung malignancies. FDG PET/CT also facilitates the evaluation of periosteal metabolic activity, often revealing increased FDG or NaF18 uptake along the periosteum in secondary HOA linked to malignancy. This uptake likely reflects active periostitis due to inflammatory or neoplastic processes. While idiopathic or non-malignant causes of HOA may present with similar imaging features, further comparative studies are needed to clarify differences in FDG uptake patterns across various etiologies [83,161,162].

Patients with suspected paraneoplastic vasculitis benefit from FDG PET/CT because of its ability to evaluate vessel wall inflammation and also detect underlying hypermetabolic malignancies [163,164,165]. This dual capability supports both the assessment of vasculitis activity and the distinction between paraneoplastic and primary autoimmune forms [164,166,167].

In paraneoplastic leukemoid reaction (PLR), FDG PET/CT has proven useful in identifying hypermetabolic tumors; for example, increased FDG uptake has been detected in cases of liposarcoma-associated PLR [97].

In VIPoma syndrome, Ga-68 DOTATATE PET/CT imaging can localize VIPomas and contribute to accurate tumor localization and staging. This imaging modality is a fundamental tool for surgical planning and assessment of eligibility for peptide receptor radionuclide therapy (PRRT) [168].

### 4.10. General Limitations of PET/CT and Tracer Selection Considerations

While FDG PET/CT has demonstrated substantial value in the detection of malignancies underlying PNSs, certain limitations should be considered in clinical practice. False-positive results may occur when FDG uptake reflects inflammatory or infectious processes rather than neoplasia, including sarcoidosis, tuberculosis, or post-radiotherapy changes, potentially leading to unnecessary invasive procedures [11,12]. For example, intense FDG uptake in mediastinal lymph nodes has been reported in suspected PNS cases but was ultimately attributable to benign granulomatous disease [11].

False negatives are equally important: well-differentiated neuroendocrine tumors, phosphaturic mesenchymal tumors, and some prostate carcinomas often have low glycolytic activity, rendering them inconspicuous or undetectable on FDG PET/CT [12,54,80]. In these scenarios—particularly when clinical suspicion remains high—somatostatin receptor-targeted PET tracers such as ^68^Ga-DOTATATE, DOTATOC, or DOTANOC may offer superior sensitivity for SSTR-expressing lesions [21,54]. In ACTH-secreting carcinoid tumors causing paraneoplastic Cushing’s syndrome, for example, ^68^Ga-DOTATATE PET/CT has successfully localized lesions that FDG PET/CT failed to detect [54].

When PET/CT findings are equivocal, integration with MRI, targeted antibody testing, or serial follow-up imaging can improve diagnostic accuracy and ensure timely initiation of appropriate therapy. Understanding these limitations and selecting the optimal tracer based on suspected tumor biology are critical to maximizing the clinical utility of PET/CT in the PNSs workup.

## 5. Summary of Diagnostic Utility of FDG PET/CT in PNSs

FDG PET/CT has demonstrated its ability to identify malignancies associated with PNSs before they are apparent on standalone anatomical imaging methods. FDG PET/CT’s whole-body imaging enables the detection of occult malignancies across multiple organ systems, a critical advantage given the systemic nature of PNSs. Studies consistently report FDG PET/CT’s ability to identify tumors otherwise not detected on anatomic imaging, particularly in neurological PNSs [8,21,104,144,169]. Sheikhbahaei et al. [21] conducted a meta-analysis of 21 studies including 1,293 patients with suspected paraneoplastic neurological syndromes (PNSs) and found that FDG PET/CT had a pooled sensitivity of 81% and specificity of 88% for detecting underlying malignancy. While specific pooled values for standalone CT or MRI were not provided, five comparative studies in their review indicated that conventional imaging modalities, such as CT and MRI, demonstrate a sensitivity ranging from 30% to 82% and specificity between 71% and 100%. These findings suggest that FDG PET/CT generally offers superior diagnostic accuracy compared to standalone CT or MRI in this clinical setting. In a cohort of 99 suspected PNS cases, Bresler et al. [170] documented 83% sensitivity and 94% specificity for FDG PET/CT, outperforming CT, which shows a sensitivity of 50% and a specificity of 100%. Vaidyanathan et al. [169] reported 100% sensitivity and 82% specificity of FDG PET-CT in a sample of 68 patients with paraneoplastic neurological disorders. Table 3 summarizes the number of patients, sensitivity, specificity, prevalence, and conditions studied in various studies using FDG PET/CT.

## 6. Clinical Impact on Patient Management

Early detection of malignancies through PET/CT imaging facilitates prompt oncologic intervention to decrease tumor burden and relieve neurological symptoms associated with PNSs [7]. In McKeon et al.’s study, PET/CT scans identified cancer in 18% of patients with suspected PNSs, enabling focused therapeutic approaches. Notably, cancer remission was achieved in seven patients, and five experienced sustained neurological improvements (median follow-up: 11 months, range: 2–48 months), demonstrating that PET/CT-guided malignancy detection benefits patient outcomes directly [172]. This enables the creation of personalized treatment plans, which are essential for both disease management optimization and improved long-term prognosis in patients with PNSs [7].

PET/CT proves its utility by differentiating between active tumors and post-treatment fibrosis with excellent predictive accuracy for detecting remaining disease. MRI serves as a primary imaging tool, but PET/CT offers superior metabolic imaging that enables more precise evaluations of recurrence and treatment response, which improves patient outcomes [177,178,179].

## 7. Conclusions

FDG PET/CT is a valuable diagnostic tool in the detection and clinical management of occult malignancies leading to PNSs. Importantly, the advantage of FDG PET/CT over standalone anatomic imaging techniques lies in its improved diagnostic accuracy by combining metabolic with structural imaging, enabling early tumor detection, leading to better outcomes in patients with PNSs. However, to overcome the limitations of PET-CT imaging, such as false-positive and false-negative findings leading to interpretation pitfalls, the accuracy of molecular imaging in conjunction with other important diagnostic tests such as antibody testing improves sensitivity and specificity of the overall diagnostic workup. Future advancements, such as hybrid PET/MRI systems which offer superior soft-tissue contrast for detecting subtle brain, spinal, and nerve lesions in neurological PNSs and enable simultaneous metabolic–anatomic imaging with lower radiation exposure and novel tracers, including radiolabeled SSTR agents, are expected to be increasingly used in PNS evaluation, particularly for ambiguous FDG-PET findings, enhancing specificity and diagnostic accuracy, and could refine diagnostic precision, optimize patient stratification, and enhance therapeutic decision-making in PNSs management.

## Figures and Tables

**Table 1:** Summary of neurologic paraneoplastic syndromes: associated antibodies, malignancies, and FDG PET/CT findings.

Syndrome	Antibodies	Associated Malignancies	FDG PET/CT Findings	Recommended PET Tracer
Paraneoplastic Limbic Encephalitis (PLE)	Anti-Hu, Anti-Ma2	SCLC, Thymoma, Breast cancer	Medial temporal lobe hypermetabolism	FDG
Paraneoplastic Cerebellar Degeneration (PCD)	Anti-Yo, Anti-Tr, Anti-CV2	Ovarian/breast cancer	Cerebellar hypometabolism	FDG
Opsoclonus-Myoclonus (OMS)	Anti-Hu, Anti-Ri, Anti-CV2	SCLC	Hypermetabolic SCLC	FDG (Neuroblastoma: also consider 123I-MIBG if available)
LEMS	Anti-VGCC	SCLC	Tumor hypermetabolism	FDG
Sensory Neuronopathy (SSN)	Anti-Hu	SCLC	Occult lung tumors	FDG

Note: SCLC = small-cell lung cancer; PET/CT = positron emission tomography/computed tomography; FDG = Fluorodeoxyglucose; CNS = central nervous system. All data presented in this table are summarized from studies cited in the corresponding subsections of the main text.

**Table 2:** Summary of Non-Neurologic Paraneoplastic Syndromes: Mechanisms, Associated Malignancies, and Role of FDG PET/CT.

Syndrome	Mechanism	Associated Malignancies	FDG PET/CT Role	Recommended PET Tracer
Cushing’s Syndrome	Ectopic ACTH secretion	SCLC, Thymoma, pancreatic neuroendocrine tumors	Tumor localization	^68^Ga-DOTATATE
Hypercalcemia	PTHrP secretion	Breast cancer, lung cancer, renal cell carcinoma, and multiple myeloma	Detect primary tumors, lytic lesions	FDG
Dermatomyositis	Anti-TIF1-γ and anti-NXP2 antibodies	Lung, ovarian, and gastrointestinal malignancies	Occult malignancy detection	FDG
Tumor-Induced Osteomalacia	FGF23 overproduction	Phosphaturic mesenchymal tumors (PMTs)	Localize PMTs	^68^Ga-DOTATATE
Leukemoid Reaction	G-CSF/GM-CSF secretion	NSCLC, Liposarcoma	Identify hypermetabolic tumors	FDG

Note: ACTH = Adrenocorticotropic Hormone; PTHrP = Parathyroid Hormone-related Protein; FGF23 = Fibroblast Growth Factor 23; PMTs = Phosphaturic Mesenchymal Tumors; G-CSF = Granulocyte Colony-Stimulating Factor; GM-CSF = Granulocyte-Macrophage Colony-Stimulating Factor; SCLC = Small-Cell Lung Cancer; NSCLC = Non-Small Cell Lung Cancer; PET/CT = Positron Emission Tomography/Computed Tomography. All data presented in this table are summarized from studies cited in the corresponding subsections of the main text.

**Table 3:** Summary of studies evaluating the diagnostic performance of FDG PET/CT in neurologic and non-neurologic paraneoplastic syndromes.

Study Reference	Number of Patients	Sensitivity (%)	Specificity (%)	Prevalence (%)	Condition Studied
Sheikhbahaei et al., [21] (meta-analysis)	1293	81	88	NA	Neurological and Non-neurological PNS
Kristensen et al. [171]	137	75	83	8.8	Neurological and Non-neurological PNS
Vaidyanathan et al. [169]	68	100	82	11.8	Neurological and Non-neurological PNS
McKeon et al. [172]	41	100	97	17.8	Neurological
Selva-O’Callaghan et al. [143]	55	67	98	NA	Dermatomyositis/polymyositis
Bannas et al. [173]	46	100	86	8.7	Neurological PNS
Schramm et al. [174]	66	100	90	13.6	Neurological PNS
Lebech et al. [175]	95	83	96	18.9	Neurological and Non-neurological PNS
García Vicente et al. [176] (meta-analysis)	793	87	86	NA	Neurological and Non-neurological PNS
Bresler et al. [170]	99	83	94	12.1	Neurological and Non-neurological PNS

## Abbreviations

The following abbreviations are used in this manuscript:

ACTH	Adrenocorticotropic Hormone
ALK	Anaplastic Lymphoma Kinase
ALL	Acute Lymphoblastic Leukemia
AlF	Aluminum Fluoride
CLL	Chronic Lymphocytic Leukemia
CNS	Central Nervous System
CRMP5	Collapsin Response Mediator Protein 5
CSF	Cerebrospinal Fluid
CT	Computed Tomography
CV2	Collapsin Response Mediator Protein 5 (also known as CV2)
DM	Dermatomyositis
DOTANOC	DOTA-1-Nal3-octreotide
DOTATATE	DOTA-DPhe1-Tyr3-Octreotate
EPO	Erythropoietin
FDG	Fluorodeoxyglucose
FGF23	Fibroblast Growth Factor 23
FNA	Fine Needle Aspiration
G-CSF	Granulocyte Colony-Stimulating Factor
GM-CSF	Granulocyte-Macrophage Colony-Stimulating Factor
HOA	Hypertrophic Osteoarthropathy
IGF	Insulin-like Growth Factor
IL	Interleukin
LEMS	Lambert-Eaton Myasthenic Syndrome
MIBG	Metaiodobenzylguanidine
MRI	Magnetic Resonance Imaging
NPV	Negative Predictive Value
NSCLC	Non-Small Cell Lung Cancer
OMS	Opsoclonus-Myoclonus Syndrome
PET	Positron Emission Tomography
PET/CT	Positron Emission Tomography/Computed Tomography
PET/MRI	Positron Emission Tomography/Magnetic Resonance Imaging
PLR	Paraneoplastic Leukemoid Reaction
PMT	Phosphaturic Mesenchymal Tumor
PPV	Positive Predictive Value
PNS	Paraneoplastic Syndromes
PCD	Paraneoplastic Cerebellar Degeneration
PLE	Paraneoplastic Limbic Encephalitis
PTH	Parathyroid Hormone
PTHrP	Parathyroid Hormone-related Protein
RT	Radiotherapy
SCLC	Small Cell Lung Cancer
SPECT	Single Photon Emission Computed Tomography
SSR	Somatostatin Receptor
SSN	Subacute Sensory Neuronopathy
SUVmax	Maximum Standardized Uptake Value
TGF	Transforming Growth Factor
TIO	Tumor-Induced Osteomalacia
VEGF	Vascular Endothelial Growth Factor
VGCC	Voltage-Gated Calcium Channel
VIP	Vasoactive Intestinal Peptide
VIPoma	Vasoactive Intestinal Peptide-Secreting Tumor
